# Comparative Performance of Large Language Models in Ophthalmology Referral Triage

**DOI:** 10.7759/cureus.102060

**Published:** 2026-01-22

**Authors:** Pedro Cardoso-Teixeira, João Alves Ambrósio, Mariana Garcia, João Chibante-Pedro, Lígia Figueiredo

**Affiliations:** 1 Ophthalmology Department, Unidade Local de Saúde Entre o Douro e Vouga, Santa Maria da Feira, PRT

**Keywords:** artificial intelligence, claude, clinical decision support, gpt, in-context learning, large language models, ophthalmology triage, perplexity pro, referral classification

## Abstract

Purpose

The aim of this study was to evaluate the classification accuracy and consistency of five advanced language model-based systems (LLMs), ChatGPT 4o, ChatGPT 5.1, Perplexity Pro, Claude Sonnet 4.5, and Claude Opus 4.1, in classifying real-world Portuguese ophthalmology referral vignettes into symptom-based categories, and to assess the effect of supervised in-context learning on model performance.

Methods

A total of 3,831 real-world, anonymized ophthalmology referral vignettes written in Portuguese and collected between January and May 2023 were submitted to each system across three independent runs. In phase one, models classified referrals into one of 16 predefined symptom-based categories using a zero-shot prompting strategy. In phase two, each system was exposed to 957 labeled examples (~20% of the dataset) through in-context learning before repeating the task. Classification accuracy, consistency, and Fleiss’ kappa agreement were calculated, with additional analysis by symptom category.

Results

Baseline classification accuracy averaged 68.7% across models, improving to 73.4% post exposure. ChatGPT 5.1 achieved the highest peak accuracy (79.5%), while ChatGPT 4o showed the largest consistency gain (from 66.8% to 93.8%) and a net improvement in 933 cases (p < 0.001). Performance exceeded 90% for common referral categories, such as diabetic screening and chronic visual loss, but was lower for rare or ambiguous complaints. Inter-run agreement, measured by Fleiss’ kappa, ranged from moderate to substantial across models (κ = 0.462-0.801), with the highest agreement observed for ChatGPT 4o.

Conclusions

Advanced LLMs demonstrated strong performance in interpreting real-world Portuguese-language ophthalmology referrals, with meaningful gains in accuracy and consistency achieved through limited supervised in-context exposure. Performance was lower for rare or ambiguous referral categories. Despite this limitation, these findings support the potential role of LLMs as scalable, low-cost triage aids, provided that human oversight and further clinical validation are ensured prior to deployment.

## Introduction

Artificial intelligence (AI) has gained increasing prominence in recent years, driven by the growing sophistication and accessibility of advanced models [1]. Among the most notable developments are large language models (LLMs), such as those developed by OpenAI (San Francisco, California, United States) and Anthropic PBC (San Francisco, California, United States), which use deep learning techniques to process and generate natural language. Tools like ChatGPT (OpenAI), Claude (Anthropic PBC), and others have attracted considerable attention for their ability to perform complex tasks across a variety of domains, including healthcare [2].

AI integration in clinical care has raised both optimism and caution. Previous studies have shown that these models can achieve high levels of accuracy in tasks such as disease diagnosis through pattern recognition and the development of predictive models based on large datasets [3-5]. However, important ethical concerns remain, particularly regarding data privacy, the potential for misinformation, and the risks associated with the unregulated use of AI in healthcare settings [6].

In ophthalmology, the accurate categorization of symptoms plays a key role in guiding diagnosis and ensuring appropriate referral. Ophthalmic conditions can present with a wide range of symptoms, such as decreased visual acuity, ocular discomfort, or visual disturbances, which may vary depending on duration and clinical context [7]. In Portugal, access to ophthalmological care requires referral from a general practitioner (GP), who must detail the patient's complaint. These referrals are then triaged and directed either to a general ophthalmology consultation or, ideally, to the relevant subspecialty [8].

The current triage system has faced criticism for inefficiencies and inconsistencies [9]. A well-executed triage process, particularly when performed by an experienced clinician, has the potential to streamline care pathways, reduce unnecessary consultations, and improve patient outcomes [8]. AI-based tools, particularly LLMs, may offer valuable support in automating and optimizing this process, a possibility that remains largely unexplored within the field of ophthalmology [10].

Unlike prior studies that have primarily relied on simulated cases, examination-style questions, or synthetic vignettes, the evaluation of language model-based systems using real-world ophthalmology referral texts generated in routine clinical practice remains largely unexplored [11]. Previous work in this field has largely focused on patient-initiated consultation questions or examination-based clinical reasoning tasks, which provide valuable insights into explanatory quality and diagnostic reasoning but do not reflect the operational constraints of real-world referral triage workflows.

Despite the importance of triage in ophthalmology, relatively few studies have examined the use of these systems in this context, and even fewer have assessed their performance using Portuguese-language referrals, which remain underrepresented in the ophthalmology triage literature [10-13]. The primary objective of this study was to evaluate and compare the classification accuracy and consistency of five advanced language model-based systems, specifically ChatGPT 4o, ChatGPT 5.1, Claude Sonnet 4.5, Claude Opus 4.1, and the Perplexity Pro platform (Perplexity AI, Inc., San Francisco, California, United States) in categorizing real-world ophthalmology referral requests into predefined symptom-based categories. Secondary objectives included assessing inter-run agreement for each model, evaluating the impact of supervised in-context exposure on classification performance, and analyzing variability in performance across referral symptom categories.

## Materials and methods

This cross-sectional observational study was conducted at Ophthalmology Department, Unidade Local de Saúde Entre o Douro e Vouga, Santa Maria da Feira, Portugal, and aimed to evaluate and compare the performance of advanced LLMs and LLM-based platforms in classifying the reasons for ophthalmology referral requests. The systems evaluated included ChatGPT 4o and ChatGPT 5.1, Claude Sonnet 4.5 and Claude Opus 4.1, as well as the Perplexity Pro platform.

Platform descriptions

ChatGPT 4o and ChatGPT 5.1 were accessed via the OpenAI ChatGPT Plus subscription. GPT-4o is optimized for speed and efficiency in multimodal tasks, while GPT-5.1 represents the most recent and powerful version of OpenAI’s model architecture as of 2025.

Claude Sonnet-4.5 and Claude Opus-4.1 are highly capable models optimized for nuanced reasoning and contextual analysis, with Opus representing the highest-performing version.

Perplexity Pro was included as a widely used clinical-facing platform rather than as a standalone LLM. It functions as an interface that provides access to multiple proprietary LLM backends through dynamic internal routing and integrates web search capabilities. Interactions were conducted via the professional user interface under default settings, and the specific backend model invoked for each request could not be uniquely identified or fixed by the user. Accordingly, results attributed to Perplexity Pro reflect platform-level behavior rather than being attributable to a single, uniquely defined model architecture.

Dataset design

The dataset was derived from a previously validated internal database containing all first-time ophthalmology consultation requests submitted by GPs to our hospital between January and May 2023. Each record included a free-text description of the reason for referral written by the referring physician. All potentially identifying information was removed prior to analysis, including patient names, dates of birth, medical record numbers, addresses, and any free-text identifiers, to ensure complete anonymization of the referral content. Requests were initially triaged and labeled by two ophthalmologists into 16 predefined symptomatic categories, based on the referral content, with final classifications reached by consensus. The data collection process is detailed elsewhere [8].

Only first-time ophthalmology referral requests containing a free-text description of the referral reason were included. Follow-up referrals, duplicate records, referrals without free-text clinical information, and entries with incomplete or unreadable content after anonymization were excluded. Records that could not be reliably assigned to one of the predefined symptom-based categories were also excluded from analysis.

The original dataset contained 4,788 entries, from which 957 were randomly selected using simple random sampling and reserved for model exposure in the second phase of the study. The remaining 3,831 cases were used as the main test set in both phases. All entries were fully anonymized and stripped of identifiable data. The final working dataset was organized in an Excel file (Microsoft Corporation, Redmond, Washington, United States) with the following columns: ID, Age, Gender, and Referral reason (free text). Table 1 shows representative examples of the records used.

**Table 1:** Examples of real anonymized GP referral texts and final triage classification Each row presents a real anonymized ophthalmology referral request from a GP, including patient age, gender, free-text reason for referral, and the final category assigned by ophthalmologists during manual triage. GP: general practitioner; M: male; F: female

ID	Age (years)	Gender	Reason for Referral	Classification
1	71	M	71-year-old patient with poorly controlled diabetes. Last retinal screening was in 2019. I request an appointment for diabetic retinopathy screening.	Diabetic retinopathy screening
2	10	F	10-year-old child complaining of difficulty seeing the board at school and the television. Visual acuity: RE 7/10, LE 8/10. I request assessment and guidance.	Chronic visual acuity loss
3	67	F	67-year-old glaucoma patient returning to ophthalmology after missing scheduled reviews for over two years. I request reassessment of intraocular pressure.	Resumption of follow-up
4	46	M	Floaters and brief flashes of light for four months.	Floaters or vitreous opacities
5	82	F	82-year-old female patient with progressive visual decline, especially when reading. Known cataracts. Referred for evaluation.	Chronic visual acuity loss
6	37	M	Type 1 diabetes; routine check requested. Visual acuity: RE 10/10, LE 10/10.	Diabetic retinopathy screening
7	63	M	Difficulty reading fine print and mild central distortion. Family history of macular disease.	Metamorphopsia or scotomas
8	29	F	Recurrent foreign body sensation and eye redness.	Ocular discomfort symptoms

Prompt structure and classification task

The input-output procedure was standardized across all tested platforms to ensure consistency and reproducibility. All interactions with the models were performed manually through their respective user interfaces, using prompts written entirely in Portuguese to match the original clinical dataset. Identical prompt wording and task instructions were used across all platforms and runs, without any model-specific adaptation. All referral vignettes were provided within a single structured prompt and processed in their original Portuguese language, without translation. Prompt entries were submitted between September and October 2025. All models were used with their default settings, without the use of any third-party plugins.

Initially, each model was presented with the classification task without any prior exposure to labeled data (zero-shot prompting strategy) [14]. The LLMs were prompted to categorize the 3,831 consultation requests presented in a structured list within a single prompt into one of 16 predefined symptomatic categories. Each case was submitted three separate times per model to evaluate both classification accuracy and output consistency. For each submission, a new chat window was initiated, the previous conversation was deleted, and all uploaded files were removed. Additionally, history and personalization settings were disabled to ensure that responses were not influenced by prior inputs. A response was considered consistent when all three outputs were identical across repetitions.

In the second phase, each model was provided with a set of 957 previously labeled examples before reprocessing the same 3,831 cases used in the baseline phase. This approach corresponds to large-scale in-context learning, in which labeled exemplars are supplied within the prompt to guide model behavior during inference, without any modification of model parameters or persistent training [15]. Although this number of examples exceeds typical few-shot settings, it was intentionally chosen to explore the upper bound of performance gains achievable through supervised in-context exposure in a real-world triage scenario [16]. The structure and wording of the prompts remained unchanged between phases, with the sole difference being the inclusion of labeled examples at the beginning of the interaction.

In both phases, the models were instructed to provide a concise classification for each case by returning only the number corresponding to the assigned category. Responses were collected in comma-separated values (CSV) format, and no additional explanatory text was allowed. Each consultation request was evaluated independently, with no reference to previous entries or outputs.

Although all prompts were presented to the language models in Portuguese, their full content, including the list of classification categories and task-specific instructions, is provided in English in Appendix A (baseline phase) and Appendix B (post-exposure phase).

Statistical analysis

Descriptive statistics were used to summarize the performance of each language model, including mean classification accuracy and consistency across the three repeated classifications. Binary consistency was defined as agreement across all three classification attempts per vignette (1 = consistent, 0 = inconsistent). Changes in consistency before and after exposure were analyzed using McNemar’s test for paired proportions. The number of vignettes that transitioned from inconsistent to consistent classification, and vice versa, was reported for each model.

Fleiss’ kappa was used to assess inter-run agreement across the three classification attempts for each model, as it is appropriate for agreement assessment involving more than two repeated ratings. Fleiss’ kappa values were interpreted according to the thresholds proposed by Landis and Koch, where values of 0.41-0.60 indicate moderate agreement, 0.61-0.80 substantial agreement, and >0.80 almost perfect agreement [17].

All analyses were performed using Excel and IBM SPSS Statistics for Windows, version 30.0 (IBM Corp., Armonk, New York, United States), and statistical significance was defined as p < 0.05. Given the large sample (n=3831), the study was adequately powered to detect changes in model consistency and accuracy.

Ethical considerations

All data used in this study were derived from a previously collected, retrospective database developed in the context of an earlier triage-related study [8], which received approval from the relevant Institutional Ethics Committee. The dataset used in the present analysis consisted exclusively of fully de-identified ophthalmology referral notes, with all potentially identifying information removed prior to analysis. The current work involved secondary analysis of anonymized data, without direct interaction with patients or access to identifiable information. In this context, no additional ethical approval was sought for the present analysis. The conduct of this research adhered to the principles of the Declaration of Helsinki.

## Results

Baseline classification performance

A total of 3,831 anonymized referral vignettes were submitted to each language model across three independent runs. In the first phase, the overall mean classification accuracy across all models was 68.7% (2,631/3,831). Among the five LLMs tested, ChatGPT 5.1 achieved the highest average accuracy (71.5%), followed by ChatGPT 4o (69.9%), Perplexity Pro (68.5%), Claude Sonnet-4.5 (68.2%) and Claude Opus-4.1 (65.6%). Accuracy per run and overall performance metrics, including Fleiss’ kappa and consistency, are summarized in Table 2.

**Table 2:** Pre-exposure performance metrics per model Accuracy and consistency for each evaluated system are shown across three independent runs prior to exposure to labeled examples. Correct and Failed indicate the number of correctly and incorrectly classified referral vignettes, respectively, out of a total of 3,831 cases per run (n (%)). Accuracy represents the proportion of correctly classified referrals per run, while Mean accuracy represents the average accuracy across the three runs. Consistency corresponds to the proportion of referral vignettes assigned to the same category in all three runs. Fleiss’ kappa (κ) quantifies inter-run agreement beyond chance. Overall values represent averages across all evaluated systems.

Chatbot	Run	Correct	Failed	Accuracy (%)	Mean Accuracy (%)	Consistency (%)	Fleiss’ Kappa (κ)
ChatGPT 4o	1	2769	1062	72.3	69.9	72.0	0.597
	2	2609	1222	68.1			
	3	2659	1172	69.4			
ChatGPT 5.1	1	2759	1072	72.0	71.5	80.0	0.730
	2	2633	1198	68.7			
	3	2833	998	73.9			
Perplexity Pro	1	2531	1300	66.1	68.5	61.0	0.337
	2	2485	1346	64.9			
	3	2851	980	74.4			
Claude Sonnet 4.5	1	2815	1016	73.5	68.2	59.0	0.507
	2	2756	1075	71.9			
	3	2270	1561	59.3			
Claude Opus 4.1	1	2772	1059	72.4	65.6	59.8	0.538
	2	2345	1486	61.2			
	3	2423	1408	63.2			
Overall					68.7	66.4	

ChatGPT 5.1 showed the highest consistency (80.0% of 3,831 referrals consistently classified across runs) while Perplexity Pro showed the lowest (61.0%). Overall, the models exhibited a mean consistency of 66.4%. Inter-run agreement ranged from moderate to substantial, with ChatGPT 5.1 again demonstrating the highest agreement (κ = 0.730).

Effect of in-context learning on accuracy and consistency

**Figure 1:**
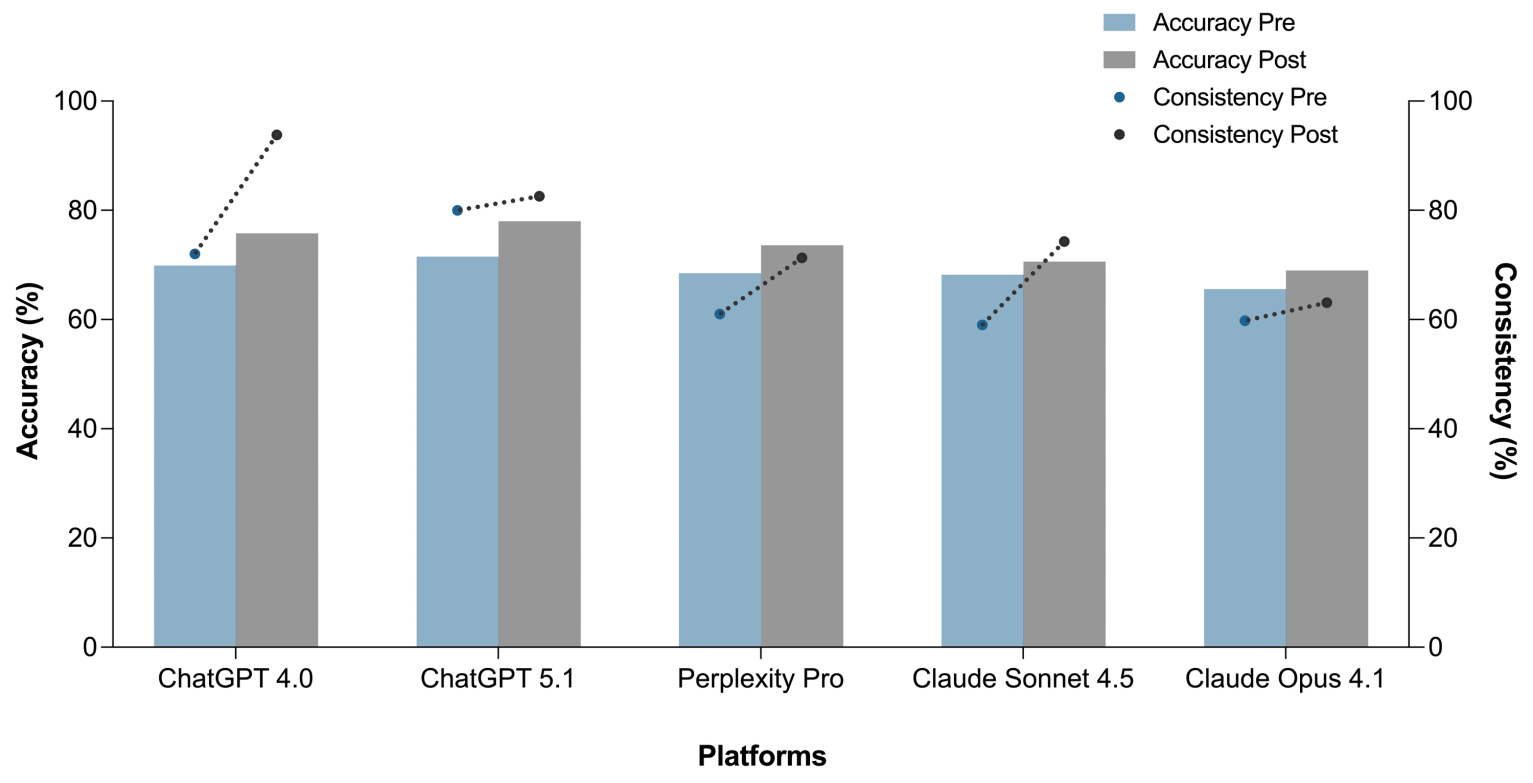
Accuracy and consistency of each LLM before and after exposure to labeled examples Bar plots represent the mean classification accuracy (left y-axis), and lines with dots represent the mean consistency across three runs (right y-axis) for each LLM. Pre- and post-exposure results are shown side by side. LLM: large language model

Following exposure to a labeled reference set in the second phase, all models showed improved performance, with an overall accuracy of 73.4%. The greatest relative improvement was observed in Claude Sonnet 4.5, which increased its best-run accuracy by +16.2 percentage points (pp). In contrast, ChatGPT 5.1 showed the smallest improvement (+5.6 pp). ChatGPT 4o reached a mean accuracy of 75.8%, with a peak of 76.7% (2,938/3,831) in a single run and a Fleiss’ kappa of 0.801. However, the highest single-run accuracy overall was achieved by ChatGPT 5.1, reaching 79.5%. Detailed post-exposure results are presented in Table 3. All other models also improved: Perplexity Pro reached a mean accuracy of 73.6%, Claude Sonnet 4.5 reached 70.6%, and Claude Opus 4.1 reached 69.0%. Consistency increased across all platforms, most notably in ChatGPT 4o, which achieved 93.8% consistency after exposure. These changes are summarized in Figure 1.

**Table 3:** Post-exposure performance and gains per system Classification performance of each system after exposure to 957 labeled referral examples. Correct and Failed indicate the number of correctly and incorrectly classified referral vignettes, respectively, out of 3,831 cases per run [n (%)]. Accuracy represents per-run classification accuracy, and Mean accuracy represents the average accuracy across the three runs. Δ Accuracy indicates the absolute change in mean accuracy (percentage points) compared with the pre-exposure phase. Consistency reflects identical categorization across all three runs, and Δ Consistency represents the absolute change relative to pre-exposure values. Fleiss’ kappa (κ) quantifies inter-run agreement beyond chance.

Chatbot	Run	Correct	Failed	Accuracy (%)	Mean Accuracy (%)	Δ Accuracy (%)	Consistency (%)	Fleiss’ Kappa	Δ Consistency (%)
ChatGPT 4o	1	2938	893	76.7	75.8	+6.9	93.8	0.801	+21.8
	2	2939	892	76.7					
	3	2833	998	73.9					
ChatGPT 5.1	1	3029	802	79.1	78.0	+3.9	82.6	0.693	+2.6
	2	3047	784	79.5					
	3	2889	942	75.4					
Perplexity Pro	1	2948	883	77.0	73.6	+5.1	71.3	0.462	+10.2
	2	2708	1123	70.7					
	3	2800	1031	73.1					
Claude Sonnet 4.5	1	2599	1232	67.8	70.6	+2.4	74.3	0.687	+15.3
	2	2894	937	75.5					
	3	2625	1206	68.5					
Claude Opus 4.1	1	2749	1082	71.8	69.0	+3.4	63.1	0.579	+3.3
	2	2380	1451	62.1					
	3	2804	1027	73.2					
Overall					73.4	+ 4.3	77.0		+10.6

A binary analysis was conducted to evaluate changes in classification consistency (from inconsistent to consistent, i.e., 0 → 1, and the reverse). ChatGPT 4o achieved the greatest net improvement, with 933 cases improving and only 97 declining, yielding a significant result (p < .001). All models showed statistically significant improvements in consistency based on McNemar’s test, as shown in Table 4. Overall, consistency gains corresponded to changes in hundreds of referral vignettes across models.

**Table 4:** Changes in classification consistency before and after exposure to labeled examples Number of referral vignettes showing improved consistency (transition from inconsistent to consistent classification; 0 → 1) or decreased consistency (consistent to inconsistent; 1 → 0) after exposure. N represents the total number of evaluated referral vignettes (n = 3,831). Statistical significance of changes in paired proportions was assessed using McNemar’s chi-square test (χ²), with corresponding p-values reported.

Model	Improved Cases (0 → 1)	Decreased Cases (1 → 0)	N	χ²	p-value
ChatGPT 4o	933	97	3831	676.92	< 0.001
ChatGPT 5.1	556	454	3831	15.04	0.001
Perplexity Pro	758	361	3831	99.72	< 0.001
Claude Sonnet 4.5	848	262	3831	253.04	< 0.001
Claude Opus 4.1	759	633	3831	9.08	0.003

Performance by symptom category 

**Figure 2:**
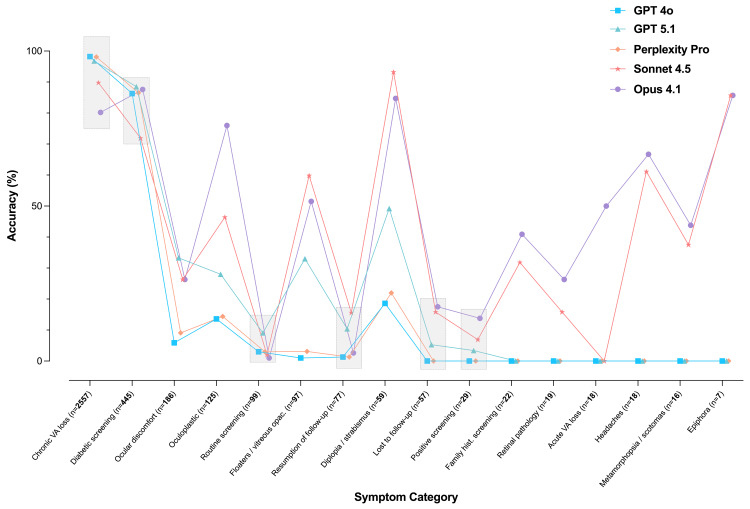
Classification accuracy per symptom category for each language model (best-performing run after exposure shown) Accuracy for each LLM is shown across the 16 classification categories. Only the best-performing run after exposure is represented. Categories are ordered from most to least frequent. Gray-shaded bars indicate symptom categories where accuracy differences between models were ≤20%, suggesting similar performance. LLM: large language model

For the analysis of per-category classification accuracy, only the best-performing run of each model after exposure to labeled examples was considered. Classification accuracy varied substantially across LLMs and symptom categories (Figure 2). Performance was highest in referrals related to chronic visual acuity loss, diabetic screening, and ocular discomfort, with several models exceeding 90% accuracy. In contrast, accuracy was consistently low in underrepresented categories such as acute visual loss, headaches, and family history screening. Five categories demonstrated comparable performance across models, with accuracy differences not exceeding 20%.

## Discussion

This study is the first to assess the performance of LLMs using real-world, anonymized ophthalmology triage data. Our findings show that current LLMs already demonstrate promising capabilities in interpreting free-text referral vignettes, with a baseline mean classification accuracy of 68.7%, increasing to 73.4% after exposure. These results are comparable to similar studies across medical domains, typically reporting accuracies in the 50-70% range [18,19]. Notably, even with a basic and standardized prompting approach, the models exhibited substantial pattern recognition ability, underscoring their potential value in clinical settings where structured data are limited or unavailable.

Prior ophthalmology-related LLM studies, including evaluations such as those by Huang et al., have predominantly focused on patient-facing question answering or examination-style reasoning tasks under controlled conditions [11,20]. In contrast, the present work evaluates model performance in an operational triage context using authentic referral texts generated in routine primary care. This distinction is relevant, as real-world referrals are often brief, heterogeneous, and non-standardized, and require consistent category assignment for workflow routing rather than narrative clinical explanations [21]. Despite these inherent challenges, the LLMs evaluated in our study demonstrated robust performance, achieving high accuracy and consistency across a large set of real-world referral vignettes.

Ophthalmology triage represents an ideal application field for LLM implementation. Our study assessed whether LLMs could categorize referrals into one of 16 symptom-based groups based solely on free-text inputs. While this classification task is relatively modest, it may serve as a foundation for more advanced applications, such as predictive tools that estimate the need for diagnostic tests (e.g., optical coherence tomography, perimetry) based on age, sex, and referral content, or models that flag cases likely to require prioritized specialist evaluation (e.g., for glaucoma) based on family history or past treatments [22,23]. Although these projects may initially seem ambitious, the vast amount of healthcare data available makes them increasingly feasible. In future developments, such systems might even assist in suggesting the appropriate timeliness of appointments or the urgency level for scheduling, provided that such recommendations remain under clinical oversight.

Our study also demonstrated that exposure to labeled examples through in-context learning can significantly enhance model performance [20,24]. After exposure to 957 randomly selected labeled examples (approximately 20% of the dataset), all models showed measurable gains. Notably, ChatGPT 4o improved its accuracy from 69.9% to 75.8% (+6.9%). While such improvements may appear modest, in clinical practice, they can translate into hundreds of correctly triaged patients monthly in high-volume settings, potentially enabling earlier specialist referral and reducing waiting times. Importantly, these performance gains were achieved using simple, standardized prompts rather than advanced prompt engineering techniques, supporting the feasibility of real-world deployment in routine ophthalmology triage workflows [14].

Ophthalmology referrals are often incomplete, contributing to inaccurate triage and longer wait times [21]. Interpreting these referrals requires clinical expertise and time, placing a burden on healthcare systems. In our service, directed subspecialty triage by experts occurs in 16-18% of cases (internal service data), improving patient flow but demanding significant human resources [8]. LLMs offer a scalable alternative, capable of processing large volumes of referrals rapidly [25]. With future integration into hospital IT systems, they could access patient histories, emergency visits, and diagnostic results. While clinicians currently synthesize this information through time-consuming chart reviews, LLMs could automate much of this process, potentially improving speed and consistency while reducing delays in patient care.

Consistency across LLMs and model divergence

Model consistency was high overall (mean 77% post-exposure), suggesting a modest, reliable repeatability in LLM outputs. However, consistency alone does not fully capture model robustness. Fleiss’ kappa, which adjusts for chance agreement, provided additional insight: while ChatGPT 5.1 showed both high consistency (80.0%) and substantial agreement (κ = 0.730), Claude Opus 4.1 exhibited only moderate agreement (κ = 0.538) despite 63.1% consistency. These discrepancies indicate that models may be confidently consistent in incorrect or suboptimal responses, highlighting the importance of measuring both repeatability and correctness.

Consistency improved across all platforms following exposure to labeled examples, suggesting that supervised in-context exposure enhances output stability in repeated classifications. This effect is particularly relevant in triage settings, where consistent routing decisions are critical for clinical safety and workflow reliability. However, the magnitude of consistency gains varied between models, likely reflecting differences in underlying architectures and training paradigms that influence how contextual information is incorporated during inference [26]. In ophthalmology, where symptom-based referrals frequently overlap across subspecialties and clinical trajectories, vignette-level error analyses would require case-by-case inference pipelines and structured error annotation frameworks, which remain important directions for future work [21,25].

While the overall trend was positive, we observed important variation in performance across both models and referral types. Models achieved >90% accuracy in common, well-structured complaints (e.g., chronic visual loss or diabetic screening), but struggled with rare or ambiguous entries (e.g., family history, headache, acute vision changes). These weaknesses are consistent with lower training exposure to less frequent categories, fewer recurring linguistic patterns, and ambiguity in natural language input [27]. Additionally, a portion of the misclassifications likely resulted from errors in the original referral text, from de-identification procedures used during anonymization, and from the inherent uncertainty of symptom-based human triage, which does not represent a definitive diagnostic ground truth. The probabilistic nature of LLMs means rarer categories inherently present more risk for divergence [16]. Subgroup analyses by symptom category revealed performance gaps of up to 30-40% in rare classes. From a clinical standpoint, misclassifications involving rare categories with potential urgency (such as acute visual loss) may carry greater consequences than errors within non-urgent or overlapping symptom groups, reinforcing the need for human oversight. This important distinction is not captured by overall accuracy metrics, which treat all misclassifications as equivalent. Because the classification framework was based on presenting symptoms rather than diagnostic labels, individual referral categories often overlapped in clinical presentation, which limited more granular performance stratification beyond the predefined symptom-based categories. Given this, a realistic ceiling for current free-text-based LLM triage accuracy may sit around 90%, with further improvements requiring integration of additional structured data.

Platform performance and selection

To ensure maximum capability, we used paid versions of each platform, which enabled bypassing input and output restrictions and leveraged enhanced reasoning features. As supported by the literature, paid LLMs typically outperform their free counterparts in complex reasoning tasks [28]. Despite using advanced models, some features were non-configurable, such as temperature and token limits, which may have impacted results [16]. Custom training pipelines, while more flexible, were beyond the scope of this study.

Across all tested LLMs, average processing times for batch input were under three minutes, demonstrating excellent speed for clinical application. However, some platforms required prompt repetition due to initial misinterpretation of bulk instructions, especially regarding CSV output formatting. Other LLMs were initially tested, such as Google Gemini (Google LLC, Mountain View, California, United States), Copilot (Microsoft Corporation), DeepSeek (Hangzhou DeepSeek Artificial Intelligence Basic Technology Research Co., Ltd., Hangzhou, Zhejiang, China), Grok (X.AI Corp., Palo Alto, California, United States), Qwen (Alibaba Group Holding Limited, Hangzhou, Zhejiang, China) but were excluded after pilot phases due to poor prompt comprehension, low performance, or operational issues, such as extended processing times or inability to output structured results.

Limitations

Several limitations must be acknowledged. First, our models only had access to de-identified free-text referral reasons. Real-world triage often considers multimodal data, including patient history, diagnostic imaging, and laboratory results, which are often decisive in human decision-making. Our study did not simulate these conditions. Second, the bulk triage setup, where the entire dataset was submitted to the LLMs simultaneously as a single input rather than case-by-case, may have limited our ability to detect model inconsistencies or hallucinations (i.e., fabricated or spurious outputs) and may introduce additional sources of variability, including formatting drift or reduced attention to intermediate content in long prompt contexts [29]. While individual case evaluation would have allowed for more granular error detection, this approach was chosen to approximate real-world, high-volume triage scenarios and assess scalability. Because all interactions were conducted through proprietary user interfaces rather than fixed application programming interface (API) endpoints, exact one-to-one reproducibility cannot be guaranteed due to potential backend updates or undocumented platform-level behaviors.

Another limitation is that both the referral vignettes and all prompts were processed in Portuguese, a language for which fewer ophthalmology triage evaluations have been published [30]. While results were strong, performance may differ across languages, particularly those with greater representation in training datasets, and may be influenced by language-specific model behavior [31], which was not assessed in this study. Lastly, although we used a structured and optimized prompt, this may not reflect how referrals are handled in typical clinical environments. Real-world deployments would likely require user-friendly interfaces and standardized prompts.

## Conclusions

LLMs show strong potential as supportive tools for ophthalmology triage. They achieved high accuracy and consistency when classifying real-world referral vignettes, with further improvement after exposure to labeled examples. Performance gains following limited supervised in-context exposure suggest a practical mechanism to enhance real-world utility without parameter-level training.

These results position LLMs as highly scalable, low-cost triage aids with immediate applicability in digitally mature health systems. Domain‑specific refinement and integration of richer clinical context will be essential before clinical deployment, along with validation against human triagers. Future research should assess the time- and cost‑efficiency of LLM-assisted triage, test performance across different languages, and explore their use in prioritization algorithms and predictive care pathways.

## Appendices

Appendix A: prompt used in phase 1 (baseline classification)

You are an ophthalmologist responsible for screening hospital ophthalmology consultation requests in Portugal. In the National Health Service (SNS), patients must first visit their general practitioner (GP) and report their eye-related issue. The GP may then decide to submit an electronic request for a hospital ophthalmology consultation, specifying the reason for the referral. These requests are then reviewed by an ophthalmologist and directed accordingly. I will provide you with consultation requests submitted between January and May 2023, organized in an Excel file. Your task is to categorize these requests to support the triage workflow. Each row contains: request number, patient age, patient gender, reason for referral described by the GP. Your goal is to classify the reason for referral into one of the following 16 categories: 1. Acute visual acuity loss: Decreased visual acuity lasting less than 3 months. 2. Chronic visual acuity loss: Decreased visual acuity lasting more than 3 months or no mention of duration. 3. Diabetic retinopathy screening. 4. Family history screening. 5. Routine screening: Only if the patient requests an ophthalmologic check-up without symptoms or known pathology, and does not fit into another screening type. 6. Ocular discomfort symptoms. 7. Diplopia or strabismus. 8. Floaters or vitreous opacities. 9. Metamorphopsia or scotomas. 10. Oculoplastic: Includes cases such as chalazion, eyelid masses, pterygium, or ptosis. 11. Lost to follow-up. 12. Resumption of follow-up. 13. Retinal pathology: Presence of any retinal condition. 14. Positive visual screening: Indicates a positive result in a previous pediatric visual screening. 15. Headaches. 16. Epiphora (excessive tearing). Rules: - If more than one reason is described, classify based on the first reason mentioned. - For each row in the file, provide a concise response (only the category number) in a new column, in CSV format. - No response may be left blank: you must always assign a triage category. - Each request must be evaluated independently, without considering previous entries. Expected Output: Your output should contain only the assigned category number for each request, along with the corresponding row number. Do not include any additional information.

Appendix B: prompt used in phase 2 (post-exposure classification)

You are an ophthalmologist responsible for screening hospital ophthalmology consultation requests in Portugal. In the National Health Service (SNS), patients must first visit their general practitioner (GP) and report their eye-related issue. The GP may then decide to submit an electronic request for a hospital ophthalmology consultation, specifying the reason for the referral. These requests are then reviewed by an ophthalmologist and directed accordingly. I will provide you with consultation requests submitted between January and May 2023, organized in an Excel file. Your task is to categorize these requests to support the triage workflow. Each row contains: request number, patient age, patient gender, reason for referral described by the GP. Your goal is to classify the reason for referral into one of the following 16 categories: 1. Acute visual acuity loss: Decreased visual acuity lasting less than 3 months. 2. Chronic visual acuity loss: Decreased visual acuity lasting more than 3 months or no mention of duration. 3. Diabetic retinopathy screening. 4. Family history screening. 5. Routine screening: Only if the patient requests an ophthalmologic check-up without symptoms or known pathology, and does not fit into another screening type. 6. Ocular discomfort symptoms. 7. Diplopia or strabismus. 8. Floaters or vitreous opacities. 9. Metamorphopsia or scotomas. 10. Oculoplastic: Includes cases such as chalazion, eyelid masses, pterygium, or ptosis. 11. Lost to follow-up. 12. Resumption of follow-up. 13. Retinal pathology: Presence of any retinal condition. 14. Positive visual screening: Indicates a positive result in a previous pediatric visual screening. 15. Headaches. 16. Epiphora (excessive tearing). Rules: - If more than one reason is described, classify based on the first reason mentioned. - For each row in the file, provide a concise response (only the category number) in a new column, in CSV format. - No response may be left blank: you must always assign a triage category. - Each request must be evaluated independently, without considering previous entries. - You have the document ”testing excel", in which 957 triages have already been performed and each request has an assigned category. Use that as a basis for learning. Expected Output: Your output should contain only the assigned category number for each request, along with the corresponding row number. Do not include any additional information.
